# Gastrointestinal neoplasia in patients with inflammatory bowel disease: Opportunities to enhance preventative strategies

**DOI:** 10.1002/jgh3.12193

**Published:** 2019-05-14

**Authors:** Edward Young, Matthew Lawrence, Michelle Thomas, Jane Andrews

**Affiliations:** ^1^ Royal Adelaide Hospital, Department of Gastroenterology and Hepatology, IBD Service Royal Adelaide Hospital Adelaide South Australia Australia; ^2^ Colorectal Surgery Unit Royal Adelaide Hospital Adelaide South Australia Australia; ^3^ University of Adelaide Adelaide South Australia Australia

**Keywords:** colorectal cancer: clinical research, colorectal cancer: epidemiology and surveillance, IBD: clinical trials, neoplasia, prevention and chemoprevention

## Abstract

**Background and Aims:**

Gastrointestinal (GI) adenocarcinoma, especially colorectal cancer (CRC), is a devastating complication of inflammatory bowel disease (IBD). We sought to examine the role of chronic inflammation and other possible predictors of the development of CRC, as well as assess as yet unexamined factors such as psychological comorbidity and engagement in care.

**Methods:**

This study included all patients involved in a tertiary hospital IBD service diagnosed with CRC between 2007 and 2017. Reports from histological specimens were assessed, and all those with adenocarcinoma, high‐grade dysplasia (HGD), or multifocal low‐grade dysplasia (LGD) originating within IBD‐affected mucosa were included in the study.

**Results:**

A total of 32 patients were included in the study (17 with adenocarcinoma and 15 with HGD/multifocal LGD). The majority had a duration of disease >20 years. Eleven patients (34%, CI 20–52%) had previous disease‐related surgery, and 16 (50%, CI 34–66%) had multiple previous disease‐related admissions. Thirteen patients (62%, CI 41–79%) had >50% of CRP results higher than 8 mg/L. Psychiatric comorbidities were common, with 19 patients (59%, CI 42–74%) having a psychiatric comorbidity or poor engagement in treatment.

**Conclusion:**

In this cohort, we have highlighted poor engagement, hesitation to up‐titrate therapy when indicated, and psychological comorbidities as likely contributors to poor disease control and development of GI adenocarcinoma. Based on our data, these easily identifiable clinical care factors should not be overlooked when addressing IBD‐related GI malignancy prevention. Additional research is required to assess a direct causal relationship, but this study would support the incorporation of psychology services into IBD clinics.

## Graphical abstract

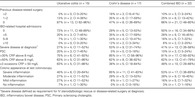

Gastrointestinal adenocarcinoma, especially colorectal cancer (CRC), is a devastating complication of inflammatory bowel disease (IBD). This study highlights poor engagement, hesitation to up‐titrate therapy when indicated, and psychological comorbidities as new risk factors of which clinicians should be aware.

## Introduction

Gastrointestinal (GI) adenocarcinoma, particularly colorectal cancer (CRC), is a devastating complication of inflammatory bowel disease (IBD), accounting for between 15 and 20% of IBD‐related mortality.^1^, ^2^, ^3^, ^4^ The incidence, although variably reported, is believed to be as high as 18% in ulcerative colitis (UC) and 8.3% in Crohn's disease (CD) after a disease duration of 30 years.^5^, ^6^ The median age of onset of CRC in IBD is as young as 43 years, up to 15–20 years earlier than that of sporadic CRC.^5^ The prognosis is poor, with an overall 5‐year survival of 33–55% for UC‐associated and 18–46% for CD‐associated CRC.^7^ IBD patients are otherwise considered to have a normal life expectancy, and therefore, efforts to prevent potentially avoidable cancers are vital.^8^, ^9^

Many larger‐scale studies have assessed risk factors of CRC in IBD, as well as prevention and detection strategies—often at a population level. The aim of our study was to analyze our cohort of patients who developed GI adenocarcinoma in the context of IBD in greater clinical detail. We aimed to assess known risk factors for GI adenocarcinoma in addition to evaluating other unexamined factors, such as psychological comorbidity and engagement in care, given the large psychological burden of IBD.

Our study included all patients known to the Royal Adelaide Hospital IBD service between 2007 and 2017. Retrospective analysis of prospectively collected data was undertaken. We reviewed our cohort to identify and examine clinical data from all cases of GI adenocarcinoma and high‐grade dysplasia (HGD) or multifocal low‐grade dysplasia (LGD) originating within IBD‐affected areas.

## Results

A total of 17 patients with adenocarcinoma and 15 patients with HGD/multifocal LGD were included (Table 1). Of the patients with adenocarcinoma, 47% (*n* = 8, CI 26–69%) had UC, and 53% (*n* = 9, CI 31–74%) had CD. There was no significant difference between males and females. Median age at diagnosis was 55 years for adenocarcinoma and 54 years for HGD/multifocal LGD. Ileocolonic disease was the most common phenotype in patients with CD and pancolitis the most common in UC (Table 2). There were no cases of GI adenocarcinoma or HGD/multifocal LGD in UC patients with isolated proctitis. Perianal disease was present in nearly half (47%; *n* = 8, CI 26–69%) of those with CD.

**Table 1:** Demographics data—gastrointestinal (GI) adenocarcinoma and high‐grade dysplasia (HGD)/multifocal low‐grade dysplasia (LGD)

	All IBD	Ulcerative colitis	Crohn's disease
Total	32	47% (*n* = 15, CI 31–64%)	53% (*n* = 17, CI 36–69%)
Gender			
Male	63% (*n* = 20, CI 45–77%)	67% (*n* = 10, CI42‐85%)	59% (*n* = 10, CI 36–78%)
Female	37% (*n* = 12, CI 23–55%)	33% (*n* = 5, CI 15–58%)	41% (*n* = 7, CI 22–64%)
Age at diagnosis			
<40	22% (*n* = 7, CI 11–39%)	33% (*n* = 5, CI 15–58%)	12% (*n* = 2, CI 3–34%)
40–59	44% (*n* = 14, CI 28–61%)	33% (*n* = 5, CI 15–58%)	53% (*n* = 9, CI 31–74%)
≥60	34% (*n* = 11, CI 20–52%)	33% (*n* = 5, CI 15–58%)	35% (*n* = 6, CI 17–59%)
SEIFA percentile			
<20	25% (*n* = 8, CI 13–42%)	13% (*n* = 2, CI 4–39%)	35% (*n* = 6, CI 17–59%)
20–39	16% (*n* = 5, CI 7–32%)	13% (*n* = 2, CI 4–39%)	18% (*n* = 3, CI 6–41%)
40–59	22% (*n* = 7, CI 11–39%)	20% (*n* = 3, CI 7–45%)	24% (*n* = 4, CI 10–47%)
60–79	22% (*n* = 7, CI 11–39%)	33% (*n* = 5, CI 15–58%)	12% (*n* = 2, CI 3–34%)
>79	16% (*n* = 5, CI 7–32%)	20% (*n* = 3, CI 7–45%)	12% (*n* = 2, CI 3–34%)

IBD, inflammatory bowel disease; SEIFA, socioeconomic indexes for areas.

**Table 2:** Montreal classification at diagnosis

	Crohn's disease		Ulcerative colitis
A1	12% (*n* = 2, CI 3–34%)	E1	0% (*n* = 0, CI 0–20%)
A2	41% (*n* = 7, CI 21–64%)	E2	40% (*n* = 6, CI 30–64%)
A3	47% (*n* = 8, CI 26–69%)	E3	60% (*n* = 9, CI 36–80%)
L1	12% (*n* = 2, CI 3–34%)		
L2	29% (*n* = 5, CI 13–53%)		
L3	59% (*n* = 10, CI 36–78%)		
B1	53% (*n* = 9, CI 31–74%)		
B2	35% (*n* = 6, CI 17–59%)		
B3	12% (*n* = 2, CI 3–34%)		
P	47% (*n* = 8, CI 26–69%)		

There were multiple markers for long‐term active inflammation in this population (Tables 3 and 4). The majority of affected patients had a disease duration of >20 years, with only one case of CRC diagnosed within 5 years of IBD diagnosis. More than half (59%; *n* = 19, CI 42–74%) had used long‐term prednisolone (defined as >12 months) at some stage in their disease course.

**Table 3:** Disease duration for gastrointestinal (GI) adenocarcinoma and high‐grade dysplasia (HGD)/multifocal low‐grade dysplasia (LGD)

	Adenocarcinoma (*n* = 17)	HGD/multifocal LGD (*n* = 15)	Combined (*n* = 32)
<5 years	6% (*n* = 1, CI 1–27%)	20% (*n* = 3, CI 7–45%)	13% (*n* = 4, CI 5–28%)
5–9 years	0% (*n* = 0, CI 0–30%)	7% (*n* = 1, CI 1–30%)	3% (*n* = 1, CI 1–16%)
10–19 years	29% (*n* = 5, CI 13–53%)	33% (*n* = 5, CI 15–58%)	31% (*n* = 10, CI 18–49%)
20–29 years	35% (*n* = 6, CI 17–59%)	27% (*n* = 4, CI 11–52%)	31% (*n* = 10, CI 18–49%)
≥30 years	29% (*n* = 5, CI 13–53%)	13% (*n* = 2, CI 4–39%)	22% (*n* = 7, CI 11–39%)

**Table 4:** Markers of inflammation—gastrointestinal (GI) adenocarcinoma and high‐grade dysplasia (HGD)/multifocal low‐grade dysplasia (LGD)

	Ulcerative colitis (*n* = 15)	Crohn's disease (*n* = 17)	Combined IBD (*n* = 32)
Previous disease‐related surgery			
>2	0% (*n* = 0, CI 0–20%)	18% (*n* = 3, CI 6–41%)	10% (*n* = 3, CI 3–24%)
1–2	13% (*n* = 2, CI 4–38%)	35% (*n* = 6, CI 17–59%)	25% (*n* = 8, CI 13–42%)
0	87% (*n* = 13, CI 62–96%)	47% (*n* = 8, CI 26–69%)	66% (*n* = 21, CI 48–80%)
IBD‐related hospital admissions			
0	73% (*n* = 11, CI 48–89%)	29% (*n* = 5, CI 13–53%)	50% (*n* = 16, CI 34–66%)
<5	20% (*n* = 3, CI 7–45%)	35% (*n* = 6, CI 17–59%)	28% (*n* = 9, CI 16–45%)
5–9	7% (*n* = 1, CI 1–30%)	24% (*n* = 4, CI 10–47%)	16% (*n* = 5, CI 7–32%)
≥10	0% (*n* = 0, CI 0–20%)	12% (*n* = 2, CI 3–34%)	6% (*n* = 2, CI 2–20%)
Severe disease at diagnosis†	27% (*n* = 4, CI 11–52%)	53% (*n* = 9, CI 31–74%)	41% (*n* = 13, CI 26–58%)
PSC	20% (*n* = 3, CI 7–45%)	0% (*n* = 0, CI 0–18%)	10% (*n* = 3, CI 3–24%)
>30% CRP above 8 mg/L	75% (*n* = 6, CI 41–93%)	85% (*n* = 11, CI 58–96%)	81% (*n* = 17, CI 60–92%)
>50% CRP above 8 mg/L	63% (*n* = 5, CI 31–86%)	62% (*n* = 8, CI 36–82%)	62% (*n* = 13, CI 41–79%)
≥3 occasions CRP > 50 mg/L	63% (*n* = 5, CI 31–86%)	38% (*n* = 5, CI 18–64%)	48% (*n* = 10, CI 28–68%)
Colonic appearance at last colonoscopy			
Severe inflammation	40% (*n* = 6, CI 20–64%)	65% (*n* = 11, CI 41–83%)	53% (*n* = 17, CI 36–69%)
Moderate inflammation	27% (*n* = 4, CI 11–52%)	29% (*n* = 5, CI 13–53%)	28% (*n* = 9, CI 16–45%)
Mild inflammation	27% (*n* = 4, CI 11–52%)	6% (*n* = 1, CI 1–27%)	16% (*n* = 5, CI 7–32%)
No inflammation	7% (*n* = 1, CI 1–30%)	0% (*n* = 0, CI 0–18%)	3% (*n* = 1, CI 1–16%)

† Severe disease defined as requirement for IV steroids/biologic rescue or disease‐related surgery at diagnosis.

IBD, inflammatory bowel disease; PSC, Primary sclerosing cholangitis.

Of the patients with CD, 11 (65%, CI 41–83%) had received treatment with biologic agents. In the overall IBD cohort, 11 (34%, CI 20–52%) had required previous disease‐related surgery, and 3 (10%, CI 3–24%) had more than two previous operations. Sixteen patients (50%, CI 34–66%) had multiple previous IBD‐related hospital admissions, with five (16%, CI 7–32%) having more than 5 and a further two (6%, CI 2–20%) having more than 10 prior admissions. Patients with CD were more likely to have been hospitalized than those with UC (73% vs. 29%, *P* = 0.01). Of those with 10 or more available C‐reactive protein (CRP) measurements, 13 patients (62%, CI 41–79%) had >50% of these measurements above 8 mg/L (upper limit of normal), with 10 patients (48%, CI 28–68%) having 3 or more measurements above 50 mg/L.

Of note was the prevalence of psychiatric comorbidities in this group prior to the diagnosis of adenocarcinoma (Table 5). Eleven patients (34%, CI 20–52%) had a formal psychiatric diagnosis of anxiety or depression prior to the diagnosis of dysplasia, with most on long‐term antidepressants. Thirteen patients (41%, CI 26–58%) had suboptimal engagement in care documented in correspondence with their GP, defined as either self‐cessation of medications or unwillingness to up‐titrate therapy when advised. In combination, 19 patients (59%, CI 42–74%) had either psychiatric comorbidity or poor engagement in treatment. As these data were gathered from case notes, this figure is likely to underestimate these factors. Socioeconomic indexes for areas (SEIFA) scores (a national government measure of socioeconomic disadvantage) appeared to be evenly distributed in both the CRC and the HGD/multifocal LGD groups (Table 1), suggesting that difficulty in engagement in care was unlikely to be predominantly due to social disadvantage.

**Table 5:** Psychosocial factors in gastrointestinal (GI) adenocarcinoma and high‐grade dysplasia (HGD)/multifocal low‐grade dysplasia (LGD)

	Ulcerative colitis (*n* = 15)	Crohn's disease (*n* = 17)	Combined IBD (*n* = 32)
Documented poor compliance/engagement	47% (*n* = 7, CI 45–70%)	35% (*n* = 6, CI 17–59%)	41% (*n* = 13, CI 26–58%)
Psychiatric diagnosis	33% (*n* = 5, CI 15–58%)	35% (*n* = 6, CI 17–59%)	34% (*n* = 11, CI 20–52%)
Combined poor engagement/psychiatric diagnosis	67% (*n* = 10, CI 42–85%)	53% (*n* = 9, CI 31–74%)	59% (*n* = 19, CI 42–74%)
Antidepressant use	27% (*n* = 4, CI 11–52%)	35% (*n* = 6, CI 17–59%)	31% (*n* = 10, CI 18–49%)
Chronic opioid use	7% (*n* = 1, CI 1–30%)	41% (*n* = 7, CI 22–64%)	25% (*n* = 8, CI 13–42%)

IBD, inflammatory bowel disease.

At their most recent colonoscopy, 17 patients (53%, CI 36–69%) had severe inflammation as defined by the presence of deep ulceration, and only one patient had no visible inflammation endoscopically (Table 4). Only nine patents with UC (60%, CI 36–80%) had been up‐titrated from 5‐aminosalicylates despite severe active inflammation, with minimal biologic use. Of the patients with CD, 65% (*n* = 11, CI 41–83%) had exposure to biologics, mostly infliximab; however, three received infliximab for less than 6 months prior to the adenocarcinoma/dysplasia diagnosis (Table 6). Long‐term outcomes were poor, with a median survival for patients with GI adenocarcinoma of 46 months for UC and 36 months for CD as of December 2017.

**Table 6:** Treatment history for gastrointestinal (GI) adenocarcinoma and high‐grade dysplasia (HGD)/multifocal low‐grade dysplasia (LGD)

Medication	Ulcerative colitis (*n* = 15)	Crohn's disease (*n* = 17)	Combined IBD (*n* = 32)
Long‐term prednisolone†	47% (*n* = 7, CI 45–70%)	71% (*n* = 12, CI 47–87%)	59% (*n* = 19, CI 42–74%)
5‐ASA	80% (*n* = 12, CI 55–83%)	71% (*n* = 12, CI 47–87%)	75% (*n* = 24, CI 58–87%)
Thiopurines	53% (*n* = 8, CI 30–75%)	76% (*n* = 13, CI 53–90%)	66% (*n* = 21, CI 48–80%)
Methotrexate	7% (*n* = 1, CI 1–30%)	18% (*n* = 3, CI 6–41%)	13% (*n* = 4, CI 5–28%)
Biologic exposure			
Total	20% (*n* = 3, CI 7–45%)	65% (*n* = 11, CI 41–83%)	44% (*n* = 14, CI 28–61%)
Infliximab	7% (*n* = 1, CI 1–30%)	53% (*n* = 9, CI 31–74%)	31% (*n* = 10, CI 18–49%)
Adalimumab	0% (*n* = 0, CI 0–20%)	24% (*n* = 4, CI 10–47%)	13% (*n* = 4, CI 5–28%)
Vedolizumab	13% (*n* = 2, CI 4–39%)	0% (*n* = 0, CI 0–18%)	6% (*n* = 2, CI 2–20%)

† Long‐term prednisolone defined as >12 months’ exposure to prednisolone at any stage.

IBD, inflammatory bowel disease.

## Discussion

Our data are novel in that they indicate new potential contributors to the development of GI adenocarcinoma and dysplasia in IBD. Psychiatric comorbidity and/or poor engagement in medical care were highly prevalent in patients presenting to a tertiary IBD service late in their disease course, and a large proportion (59%, CI 42–74%) of the service's total diagnoses of neoplasia over the last decade came from this group. Those at risk presented to the referral service after many years of poorly controlled inflammation without exposure to maximal available therapy. These data suggest that, from a community perspective, efforts should be made to better educate patients and nonspecialist IBD clinicians about the cancer risks of poor disease control. Specific attention to mental health issues and exploration of the reasons for failure of up‐titration of therapy are also warranted. These novel findings warrant investigation in a larger case–control study to evaluate whether they are indeed independent risk factors for poor disease control and the development of IBD‐associated malignancy/dysplasia.

It has been well established that chronic inflammation is the key component to the development of CRC in IBD.^10^, ^11^, ^12^, ^13^, ^14^ Many of the measured data points in this study reflected many years of severely active colonic inflammation prior to the development of adenocarcinoma. There were high rates of hospitalization and previous disease‐related surgery, repeated significantly elevated inflammatory markers, and prolonged steroid use. Interestingly, patients with UC were significantly less likely to have had previous hospital admissions despite similar biochemical and endoscopic markers of inflammation, perhaps suggesting a tendency for patients and clinicians to underestimate UC severity. Persistently elevated inflammatory markers, multiple hospitalizations, and disease‐related surgeries represent lost opportunities to recognize the failure of disease control and escalate therapy to achieve healing, thereby reducing the risk of neoplasia.

Despite the severity of inflammation, many of these patients did not have more aggressive therapy initiated. In those with UC, only nine (60%, CI 36–80%) had been up‐titrated to a thiopurine. A 2018 meta‐analysis by Zhu et al. reinforced the antineoplastic activity of thiopurine‐based immunomodulators, with an odds ratio of 0.51 for HGD and 0.55 for CRC.^15^ Rates of immunomodulator and biologic use in CD were higher, although nearly half (47% (*n* = 8, CI 26–69%) only received a biologic for 6 months or less prior to the neoplasia diagnosis. While there are few long‐term data regarding CRC risk reduction with anti‐TNF agents, a 2011 Dutch case–control study of 173 cases of IBD‐associated CRC found a significant reduction in CRC risk with the use of infliximab, with an odds ratio of 0.09.^16^, ^17^

The failure to deliver appropriate (healing) treatment may be partly a result of patient engagement factors. The high rates of anxiety and depression in our cohort likely contributed significantly to the rates of difficult engagement in treatment. Engagement issues included self‐cessation of medications, hesitancy to up‐titrate therapies, and—in one instance—declining colorectal surgical input for severe perianal disease and thus rendering the patient ineligible for anti‐TNF therapy (due to the sepsis risk). Given the chronic nature of IBD and the requirement for long‐term adherence to therapy to maintain disease control, psychological comanagement is imperative to optimize outcomes. Psychologist involvement in specialist IBD clinics should be considered as it has been shown to improve medication adherence and reduce hospital admissions.^18^

## Conclusion

Those at greatest risk of GI adenocarcinoma in IBD should be recognized in clinical care. They have extensive disease, poor disease control, prolonged steroid use, recurrent admissions, and a history of IBD surgery. In this cohort, we have, for the first time, highlighted poor engagement in care, hesitation to up‐titrate therapy when indicated, and psychological comorbidities as potential additional contributors of which clinicians should be aware.

Based on our data, the primary focus of IBD‐related GI malignancy prevention should be on these identifiable clinical care factors to reduce inflammation in the first instance and subsequently reduce the eventual development of dysplasia requiring detection. One such method for addressing these factors would be the incorporation of psychology services into IBD centers, with the aim of improving patient engagement and addressing psychiatric comorbidities.
